# Supplementary material for: Hybrid Models and Biological Model Reduction with PyDSTool
Source: PLoS Comput Biol. 2012 Aug 9;8(8):e1002628. doi: 10.1371/journal.pcbi.1002628 (PMC3415397; doi:10.1371/journal.pcbi.1002628)
Supplement: Text S4 — Complete source code for the PyDSTool package (version 0.88.120504). Includes API documentation and help files linking to web pages. This file is identical to the current public release on Sourceforge.net. (ZIP) [file pcbi.1002628.s004.zip › PyDSTool/html/PyDSTool.Variable'.Variable-class.html]

xml version="1.0" encoding="ascii"?


PyDSTool.Variable'.Variable


| Home | Trees | Indices | Help | PyDSTool |
| --- | --- | --- | --- | --- |

Package PyDSTool ::
Module Variable' ::
Class Variable

[hide private]

[frames] | no frames]

# Class Variable

source code

```
object --+
         |
        Variable
```

Known Subclasses:
:   - HybridVariable

---

One-dimensional discrete and continuous real variable class.


|  |  |
| --- | --- |
| Instance Methods | [hide private] |

|  |  |
| --- | --- |
| \_\_call\_\_(self, indepvar, checklevel=0) | source code |

|  |  |
| --- | --- |
| \_\_copy\_\_(self) | source code |

|  |  |
| --- | --- |
| \_\_deepcopy\_\_(self, memo=None, \_nil=`[``]`) | source code |

|  |  |
| --- | --- |
| \_\_del\_\_(self) | source code |

|  |  |
| --- | --- |
| \_\_getstate\_\_(self) | source code |

|  |  |
| --- | --- |
| \_\_init\_\_(self, outputdata=None, indepdomain=None, depdomain=None, name=`'``noname``'`, abseps=None, labels=None)  x.\_\_init\_\_(...) initializes x; see x.\_\_class\_\_.\_\_doc\_\_ for signature | source code |

|  |  |
| --- | --- |
| \_\_repr\_\_(self)  str(x) | source code |

|  |  |
| --- | --- |
| \_\_setstate\_\_(self, state) | source code |

|  |  |
| --- | --- |
| \_\_str\_\_(self)  str(x) | source code |

|  |  |
| --- | --- |
| \_auxfn\_getindex(self, parsinps, varname) | source code |

|  |  |
| --- | --- |
| \_auxfn\_globalindepvar(self, parsinps, t) | source code |

|  |  |
| --- | --- |
| \_auxfn\_heav(self, parsinps, x) | source code |

|  |  |
| --- | --- |
| \_auxfn\_if(self, parsinps, c, e1, e2) | source code |

|  |  |
| --- | --- |
| \_auxfn\_initcond(self, parsinps, varname) | source code |

|  |  |
| --- | --- |
| \_infostr(self, verbose=1) | source code |

|  |  |
| --- | --- |
| \_setRanges(self, abseps=None) | source code |

|  |  |
| --- | --- |
| addMethods(self, funcspec)  Add dynamically-created methods to Veriable object | source code |

|  |  |
| --- | --- |
| getDataPoints(self)  Reveal underlying mesh and values at mesh points, provided Variable is based on a mesh (otherwise None is returned). | source code |

|  |  |
| --- | --- |
| info(self, verboselevel=1) | source code |

|  |  |
| --- | --- |
| is\_continuous\_valued(self) | source code |

|  |  |
| --- | --- |
| is\_discrete\_valued(self) | source code |

|  |  |
| --- | --- |
| setDepdomain(self, depdomain, abseps=None) | source code |

|  |  |
| --- | --- |
| setIndepdomain(self, indepdomain, abseps=None) | source code |

|  |  |
| --- | --- |
| setOutput(self, outputdata, funcspec=None, globalt0=0, var\_namemap=None, ics=None, refvars=None, abseps=None)  Dynamically create 'output' method of Variable | source code |

|  |  |
| --- | --- |
| truncate\_to\_idx(self, idx) | source code |

|  |  |
| --- | --- |
| underlyingMesh(self)  Reveal underlying mesh as arrays, rather than Pointset as returned by getDataPoints method. | source code |

**Inherited from `object`**:
`__delattr__`,
`__getattribute__`,
`__hash__`,
`__new__`,
`__reduce__`,
`__reduce_ex__`,
`__setattr__`


|  |  |
| --- | --- |
| Properties | [hide private] |

**Inherited from `object`**:
`__class__`


|  |  |
| --- | --- |
| Method Details | [hide private] |

|  |  |
| --- | --- |
| \_\_init\_\_(self, outputdata=None, indepdomain=None, depdomain=None, name=`'``noname``'`, abseps=None, labels=None)  *(Constructor)* | source code |

x.\_\_init\_\_(...) initializes x; see x.\_\_class\_\_.\_\_doc\_\_ for
signature

Overrides: object.\_\_init\_\_: *(inherited documentation)*

|  |  |
| --- | --- |
| \_\_repr\_\_(self)  *(Representation operator)* | source code |

str(x)

Overrides: object.\_\_repr\_\_: *(inherited documentation)*

|  |  |
| --- | --- |
| \_\_str\_\_(self)  *(Informal representation operator)* | source code |

str(x)

Overrides: object.\_\_str\_\_: *(inherited documentation)*

|  |  |
| --- | --- |
| getDataPoints(self) | source code |

Reveal underlying mesh and values at mesh points, provided Variable is
based on a mesh (otherwise None is returned). The returned Pointset will
be time-shifted according to the Variable's current \_internal\_t\_offset
attribute.

Any pointset labels present when the variable was created will be
restored.

|  |  |
| --- | --- |
| underlyingMesh(self) | source code |

Reveal underlying mesh as arrays, rather than Pointset as returned by
getDataPoints method. If no underlying mesh is present, None is
returned.

  


| Home | Trees | Indices | Help | PyDSTool |
| --- | --- | --- | --- | --- |

|  |  |
| --- | --- |
| Generated by Epydoc 3.0.1 on Fri May 4 15:24:10 2012 | http://epydoc.sourceforge.net |
